# Evaluation of long-term outcome following therapeutic mammaplasty: the effect of wound complication on initiation of adjuvant therapy and subsequent oncological outcome

**DOI:** 10.1308/rcsann.2023.0095

**Published:** 2024-05-15

**Authors:** R Rampal, S Jones, W Hogg, B Rengabashyam, B Hogan, R Achuthan, B Kim

**Affiliations:** ^1^ The Leeds Teaching Hospitals NHS Trust, UK; ^2^ Mid Yorkshire Teaching NHS Trust, UK; ^3^ York and Scarborough Teaching Hospitals NHS Foundation Trust, UK

**Keywords:** Therapeutic mammaplasty, Adjuvant therapy, Wound complications, Oncological outcome, Oncoplastic breast surgery

## Abstract

**Introduction:**

Therapeutic mammaplasty (TM) facilitates large tumour resection while maintaining optimal aesthetic outcome. It carries higher wound complication risks, which may delay adjuvant therapy initiation. Whether this delay affects oncological outcome requires evaluation.

**Methods:**

Data were collected for consecutive patients receiving TM at the Leeds breast unit (2009–2017). A prospectively maintained database was used to determine tumour characteristics, wound complication rates, receipt of adjuvant therapy and breast cancer recurrence or death.

**Results:**

In total 112 patients (median age of 54 years) underwent 114 TM procedures. The most common histological subtypes were invasive ductal carcinoma (61.4%), invasive lobular carcinoma (13.2%) and ductal carcinoma in situ (13.2%). Of the patients, 88.2% had oestrogen receptor-positive cancer and 14% had human epidermal growth factor receptor-positive cancer; 26.3% had multifocal cancer. The median tumour size was 30mm. The median Nottingham Prognostic Index was 4.2. The local recurrence rate was 3.5% (median follow-up of 8.6 years). The 5- and 10-year disease-free survival (DFS) was 88.5% and 83.5%, and the equivalent overall survival (OS) rates were 94% and 83.5%. Wound complication rate was 23.6% (*n*=27), the commonest being wound infection (11.4%; *n*=13) and T-junction wound breakdown (10.5%; *n*=12). The median time to adjuvant therapy was 72 days (interquartile range [IQR] 56–90) for patients with wound complications, and 51 days (IQR 42–58) for those without. However, this delay did not affect DFS or OS (log-rank test; *p*=0.58 and *p*=0.94, respectively). This was confirmed on Cox regression analysis.

**Conclusion:**

Our study finding demonstrates that although wound complications after TM leads to a modest delay to adjuvant therapy, the long-term oncological outcomes were comparable with those in patients without wound complications.

## Introduction

Breast conservation surgery (BCS) techniques are widely utilised to treat breast cancer.^1^ Oncoplastic BCS techniques are well established with the aim of reducing mastectomy and re-excision rates, while minimising breast deformity.^2,3^ Therapeutic mammaplasty (TM) is a type of oncoplastic BCS that is indicated when tumour resection is greater than 20% of breast volume.^3^ TM utilises plastic surgery techniques to displace breast volume for optimal breast reshaping once cancer is resected. This is combined with a reduction of the skin envelope such as mastopexy to optimise aesthetic outcome.^4^

Short-term outcomes after TM have been well reported in terms of relatively low complication rates and comparable margin re-excision rates to standard BCS.^5,6^ Furthermore, other studies have established the long-term oncological safety of TM in terms of locoregional recurrence risks that are comparable with standard BCS or mastectomy.^7,8^

The most common type of surgical approach for TM is via a wise-pattern skin incision.^6^ This approach is commonly utilised for breast reduction surgery and has higher wound complication risks than standard BCS.^9^ The range of wound complications include T-junction (where the horizontal part of the scar meets the vertical part of the scar at the inframammary fold) wound breakdown, skin flap necrosis, nipple necrosis, wound infection, haematoma and fat necrosis. A meta-analysis of studies reporting outcomes after breast reduction surgery reported complication rates ranging from 6.5% to 22%.^10^ The international TeaM multicentre prospective cohort study similarly reported a complication rate of 23.3% for 880 patients undergoing TM procedures across 50 centres.^6^

Unlike patients undergoing breast reduction surgery, patients receiving TM will undergo further adjuvant breast cancer treatments such as radiotherapy or chemotherapy. If patients develop postoperative complications after TM, this may result in a significant delay to the initiation of adjuvant therapy.^11^ Substantial delay in the receipt of adjuvant therapy has been shown to negatively impact long-term oncological outcome. A delay of more than 8 weeks (56 days) between breast cancer surgery and receipt of radiotherapy has been shown to negatively impact locoregional recurrence risk.^12^ Similarly, a delay of more than 90 days between breast cancer surgery and initiation of chemotherapy has been shown to result in poorer survival outcomes.^13^

As stated above, TM is a safe oncological procedure but has the potential for higher wound complication risks. These complications may delay the initiation of adjuvant therapy. Although these potential issues are well documented and frequently discussed with patients in the clinic, there is a lack of published research assessing the extent of delays that may be incurred and how it might affect long-term oncological outcomes. This remains relatively unexplored in patients receiving TM, especially given the relatively recent adoption of this surgical technique. We therefore sought to evaluate the effect of wound complications on the timings to the initiation of adjuvant therapy and whether this impacted on oncological outcomes for patients receiving TM in our centre.

## Methods

This was a single-centre retrospective cohort study with data collection performed using a prospectively maintained electronic patient record system (Patient Pathway Manager [PPM]). All data extracted for the study were routinely recorded on PPM as part of standard clinical care. As per the hospital research governance protocol, no ethical approval was required. Data were collected for all consecutive patients receiving wise-pattern TM at the Leeds breast unit between 2009 and 2017. This period was chosen to enable a minimum of 3-year follow-up time to examine oncological outcome including local recurrence rate (LRR), disease-free survival (DFS) and overall survival (OS). All surgeons in the Leeds breast unit perform wise-pattern TM routinely without drain usage. Simultaneous contralateral breast reduction surgery was performed based on the discussion between the operating surgeon and stated patient preference. Nipple blood supply was maintained on a dermoglandular pedicle unless tumour proximity indicated nipple excision. All patients received prophylactic antibiotics with a single dose of intravenous co-amoxiclav at induction of anaesthesia. Patients allergic to penicillin received a single dose of intravenous teicoplanin. Decisions for intraoperative cavity shaves were based on intraoperative x-ray analysis of the resection specimen (Faxitron^™^ OR specimen radiography system). Adjuvant treatment decisions were based on the Leeds breast unit multidisciplinary team (MDT) meeting discussion outcomes.

Inclusion criteria were all patients who underwent a wise-pattern TM for unilateral or bilateral operable primary breast cancer. All invasive or non-invasive breast cancer subtypes were included. Patients either presented with symptomatic cancer or via the National Health Service breast screening programme. Patients with unifocal or multifocal cancers were included. For the latter cases, decision for TM was based on MDT meeting discussion. There was no radiology maximal tumour size cut-off for TM but this was based on tumour to breast volume ratio and whether TM was surgically feasible as assessed by the operating surgeon. Exclusion criteria were patients who had TM via any other approach (e.g. vertical scar/Benelli mammaplasty). Similarly, patients undergoing other types of oncoplastic BCS, such as chest wall perforator volume replacement, were excluded. Patients undergoing standard BCS or mastectomy with or without immediate breast reconstruction were excluded. Patients diagnosed with locoregional recurrence or those diagnosed with distant metastasis during preoperative investigations were also excluded.

Data collection comprised the following patient demographics: patient age and patient comorbidities (diabetes, smoking status and steroid use). Data on tumour characteristics included: mode of presentation (symptomatic/screening), histological tumour subtype, unifocality or multifocality, tumour size on imaging and pathology, magnetic resonance imaging use, TNM staging, Nottingham Prognostic Index score, molecular receptor status (oestrogen receptor positive [ER], progesterone receptor positive [PR] and human epidermal growth factor receptor positive [HER2]). Data collection also included tumour resection margin width, date of recurrence (locoregional/distant), date of death (related to breast cancer or due to other cause), receipt of adjuvant (or neoadjuvant) therapies and date commenced. Data on surgical factors included: date of surgery, laterality, localisation techniques for impalpable lesion, type of axillary surgery, simultaneous contralateral breast reduction, nipple excision, intraoperative cavity shave, specimen weight, further oncological surgery (involved margins or requirement for further axillary surgery) and postoperative wound complications (wound breakdown, skin flap with or without nipple necrosis, wound infection, haematoma, fat necrosis, type and date of surgery to treat complications). We classified surgical complications according to the Clavien–Dindo grading of surgical complications.^14^ In particular, T-junction breakdown was defined as a wound dehiscence requiring additional dressings (e.g. inadine) to expedite re-epithelialisation, or where surgical intervention was required to treat wound breakdown. Data on wound complications were recorded by reviewing electronic case notes (PPM). Patients were routinely seen in the surgical clinic at 14 days post-surgery with record of absence or presence of any wound complications. Case note review was performed on patients with wound complications to determine the time to resolution of wound complications. Furthermore, further case note review was performed to determine any delayed wound complications or reoperations to treat wound complications at 30 days.^6^

### Statistical analysis

Statistical analysis was performed using SPSS, v28.0. Continuous variables were presented as mean (SD) or medians (interquartile range [IQR]). Categorical variables were presented as frequency (%). Variables of interest were tested for normality of distribution using SPSS. The chi-squared test was used to test for association between categorical variables, and independent *t* tests for continuous variables. Kaplan–Meier survival analysis was used to determine DFS and OS.^15^ Multivariate Cox regression analysis was performed to explore the effect of wound complications and delay in adjuvant therapy receipt against LRR, DFS and OS. Values of *p*≤0.05 were deemed to be statistically significant.

## Results

During the study period, 112 patients underwent 114 TM procedures over 8 years (2009–2017). The median patient age was 54 years (IQR 48.2–61). With regards to patient characteristics, 10.5% were active smokers, 6% were ex-smokers, 3.5% were diabetic and 0.9% were on steroids. Some 43% of the study cohort presented with screen-detected breast cancer. In total, 26.3% of the study cohort underwent TM for multifocal breast cancer. The most common histological subtype was invasive ductal carcinoma (IDC) (61.4%; *n*=70) followed by invasive lobular carcinoma (ILC) (13.2%; *n*=15) and ductal carcinoma in situ (DCIS) (13.2%; *n*=15). With regards to molecular receptor profile of the study cohort, 88.2% (*n*=85) were ER positive, 77.4% (*n*=70) were PR positive and 14% (*n*=15) were HER2 receptor positive. The median whole tumour size was 30mm (IQR 20–40). The median Nottingham Prognostic Index score for invasive cancer was 4.2 (IQR 3.3–4.6).

With regards to postoperative TNM staging, 35 patients (31.3%) presented with T1 disease, 51 patients with T2 disease (45.5%) and 7 patients (6.3%) with T3 disease. Most patients (*n*=72; 64.3%) had negative axilla (N0). For patients with positive axilla, 31 patients (27.7%) had N1 disease, 6 patients (5.4%) had N2 disease and 2 patients had N3 (1.8%) disease. In the study cohort, 23 patients (20.2%) were treated with neoadjuvant chemotherapy.

For patients receiving TM, 16 patients (14.3%) underwent simultaneous contralateral breast reduction surgery. Forty-six patients (41%) underwent contralateral breast reduction during the follow-up phase. The remaining 50 patients (44.6%) did not to undergo contralateral breast reduction surgery. With regards to axillary surgery, sentinel lymph node biopsy (SLNB) was performed in 68 cases (59.6%), upfront axillary node clearance (ANC) performed in 25 cases (21.9%) and SLNB followed by completion ANC performed in 7 cases (6.1%). No axillary surgery was performed in 14 cases because patients underwent TM to treat DCIS (12.3%).

Further oncological surgery was performed in 17 cases (14.9%) because of insufficient tumour margin clearance; 6 patients (35.3%) underwent margin re-excision and 11 patients (64.7%) patients underwent completion mastectomy with or without immediate breast reconstruction.

**Figure 1:**
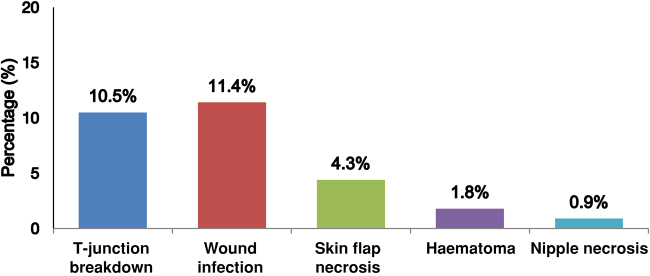
Postoperative complications of the study cohort

Postoperative complications (Figure 1) were observed in 27 patients (23.6%). Most of the wound complications (77.8%; 21/27) were Clavien–Dindo grade I or II, where patients required additional dressing therapy to expedite wound healing or antibiotics to treat wound infection. The remaining (22.2%; 6/27) complications were classified as Clavien–Dindo grade III because they required unplanned surgery to treat complications. In terms of specific complications, wound infection was the most common complication occurring in 13 patients (11.4%), followed by T-junction wound breakdown (10.5%; *n*=12). Other less-frequent postoperative complications included haematoma (1.8%; *n*=2), skin flap necrosis (4.3%; *n*=5) and nipple necrosis (0.9%; *n*=1). Table 1 provides comparison of the clinicopathological characteristic between patients who suffered from wound complications vs patients without wound complications. Both groups had comparable clinicopathological characteristics, apart from a higher rate of diabetes seen in patients with wound complications.

**Table 1:** Clinicopathological characteristics of the study cohort

Patient demographics											
	Wound complications (*n*=27)					No wound complications (*n*=87)					*p*-value
Age (median)	56					53					0.48
Smoking status (active or ex)	14.8% (*n*=4)					17.6% (*n*=15)					0.76
Diabetes	11.1% (*n*=3)					1.2% (*n*=1)					**0.01**
Steroid use	0% (*n*=0)					1.2% (*n*=1)					0.5
Tumour characteristics											
Histological subtypes	IDC	ILC	Pure DCIS	Others	Mixed IDC/ILC	IDC	ILC	Pure DCIS	Others	Mixed IDC/ILC	
	74.1% *n*=20	14.8% *n*=4	7.4% *n*=2	3.7% *n*=1	0% *n*=0	57.5% *n*=50	12.6% *n*=11	15% *n*=13	6.9% *n*=6	8% *n*=7	0.35
Molecular status	ER positive: 85.1% (*n*=23)					ER positive: 71.3% (*n*=62)					0.75
	PR positive: 74% (*n*=20)					PR positive: 57.4% (*n*=50)					0.67
	HER2 positive: 14.8% (*n*=4)					HER2 positive: 10.3% (*n*=9)					0.52
Unifocal or multifocal	Unifocal		Multifocal			Unifocal		Multifocal			
	74% (*n*=20)		26% (*n*=7)			73.6% (*n*=64)		26.4% (*n*=23)			0.95
T staging*	Tis	T1	T2	T3		Tis	T1	T2	T3	
	7.4% *n*=2	48.1% *n*=13	44.4% *n*=12	*n*=0		16.9% *n*=14	27.7% *n*=23	47% *n*=39	8.4% *n*=7		0.10
*N* staging*	N0	N1	N2	N3		N0	N1	N2	N3		
	70.4% *n*=19	18.5% *n*=5	7.4% *n*=2	3.7% *n*=1		63.9% *n*=53	31.3% *n*=26	4.8% *n*=4	1.2% *n*=1		0.51
Axillary surgery	No axillary surgery: 7.4% (*n*=2) SLNB: 63% (*n*=17) ANC: 25.9% (*n*=7) Completion ANC: 3.7% (*n*=1)					No axillary surgery: 29.9% (*n*=26) SLNB: 42.5% (*n*=37) ANC: 20.7% (*n*=18) Completion ANC: 6.9% (*n*=6)					0.084
Systemic therapies	Neoadjuvant chemotherapy: 18.5% (*n*=5)					Neoadjuvant chemotherapy: 20.6% (*n*=18)					0.98
	Adjuvant endocrine therapy: 74.1% (*n*=20)					Adjuvant endocrine therapy: 69% (*n*=60)					0.54
	Adjuvant Herceptin: 14.8% (*n*=4)					Adjuvant Herceptin: 9.2% (*n*=8)					0.41

*TNM staging not available for four patients with ‘other’ tumour types (all without complications

ANC = axillary node clearance; DCIS = ductal carcinoma in situ; ER = oestrogen receptor; HER2 = human epidermal growth factor receptor 2; IDC = invasive ductal carcinoma; ILC = invasive lobular carcinoma; PR = progesterone receptor; SLNB = sentinel lymph node biopsy

In total, 97 patients (86%) received adjuvant radiotherapy, with one patient receiving bilateral radiotherapy. We explored reasons for patients not receiving adjuvant radiotherapy after BCS. Of the remaining 15 patients (16 breasts) who did not receive radiotherapy, 6 (40%) required completion mastectomy because of insufficient margin clearance. Five patients (33.3%) had non-invasive cancer where radiotherapy was not indicated as per MDT recommendations. One patient (6.7%) declined radiotherapy, one patient (6.7%) had omission of radiotherapy because of prolonged wound complications and two patients (13.3%) were diagnosed with subsequent distant metastatic disease on postoperative staging investigations.

Adjuvant chemotherapy was administered in 41 patients (36.6%). For the study cohort, the median duration from surgery date to commencement of either adjuvant chemotherapy or radiotherapy was 54 days (IQR 44–64). This specified duration was median of 72 days (IQR 56–90) for patients who suffered from wound complications, as opposed to median of 51 days (IQR 42–58) for patients without wound complications. Therefore, there was a median delay of 21 days to the commencement of adjuvant therapy for patients with wound complications. For patients with wound complications (*n*=27), 16 (59.3%) suffered from a delay in commencement of adjuvant therapies, defined as longer than 56 days.^12^ For all 16 patients (100%), wound complication was the sole cause of delay to adjuvant therapy. For patients without wound complications (*n*=87), 20 (23%) suffered from a delay in commencement of adjuvant therapies. Of these 20 patients, 7 (35%) required further oncological surgery resulting in the delay and 4 (20%) required further decision-making time about adjuvant therapy, with no specific reason identified for the remaining 9 patients (45%).

**Figure 2:**
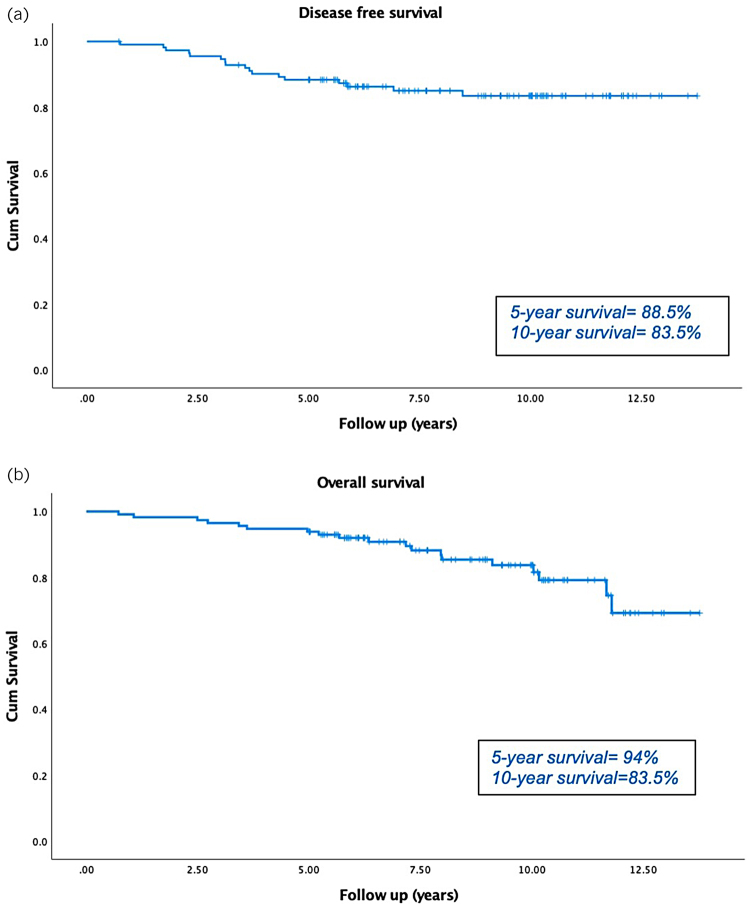
Kaplan–Meier curves showing (a) disease-free survival and (b) overall survival at 5 and 10 years. Survival time is shown in years

The LRR of the study cohort was 3.5% (median follow-up of 8.6 years; IQR 6.1–10.3). Kaplan–Meier survival analysis showed that the 5-year DFS and OS were 88.5% and 94% respectively (Figure 2). The 10-year DFS and OS were 83.5% and 83.5% respectively (Figure 2).

**Figure 3:**
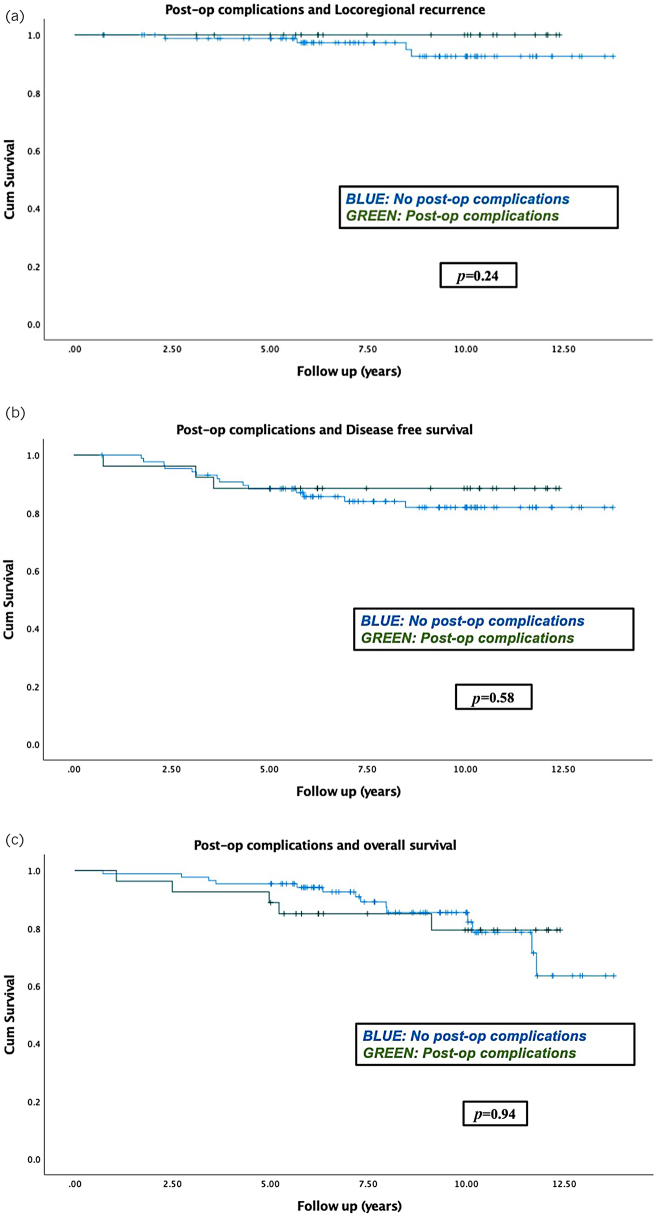
Kaplan–Meier analyses comparing the outcomes of patients with and without postoperative complications: (a) locoregional recurrence, (b) disease-free survival and (c) overall survival. No statistically significant difference was observed. Survival time is shown in years.

We then explored whether there was a difference in LRR, DFS and OS between patients with wound complications vs patients without. The Kaplan–Meier analysis (Figure 3) demonstrated that the delay in the receipt of adjuvant therapies seen in the wound complication group did not adversely impact LRR, DFS or OS (log-rank test; *p*=0.24, *p*=0.58 and *p*=0.94, respectively).

The above findings were also confirmed on multivariate Cox regression analysis, which was performed to further evaluate the log-rank test findings. The following clinicopathological covariates were examined as potentially impacting on survival outcomes: patient age, T stage, N stage, receipt of adjuvant therapy, delay in adjuvant therapy, molecular receptor profile, tumour subtype and unifocality or multifocality. The Cox regression analysis again demonstrated that the delay in the receipt of adjuvant therapies seen in the wound complication group did not adversely impact LRR, DFS or OS (*p*=0.58, *p*=0.6 and *p*=0.59, respectively).

## Discussion

Our study findings demonstrate that TM is a valuable BCS technique in being able to remove relatively larger cancers, with low long-term LRR and favourable survival outcomes. Almost half (45.5%) of the study cohort had T2 tumour (between 2 and 5cm) which may have required mastectomy. Further surgery because of incomplete margin clearance was required in 14.9% of patients and the eventual mastectomy rate of the study cohort was 9.6% (*n*=11), meaning that most patients were able to preserve their native breast while achieving favourable long-term oncological outcomes. A recent Swedish cohort study of almost 50,000 patients has highlighted that for patients with T1–2, N0–2 invasive breast cancer, those receiving BCS and radiotherapy had better survival than those receiving mastectomy, irrespective of receipt of radiotherapy.^16^ Therefore, advanced BCS techniques such as TM should continue to be utilised to provide BCS surgery options where feasible. Our 14.9% reoperation for incomplete margin clearance is similar to the 14.7% reported by the international TeaM multicentre cohort study which recruited 880 patients.^6^

Our LRR was 3.5% with a median follow-up of 8.6 years, which is similar to the LRR of 3.2% reported by De La Cruz who performed a systemic review on 6,011 patients undergoing TM (mean follow-up of 50.5 months).^17^ Therefore, our study finding continues to support the utilisation of TM and adds to the growing evidence of its oncological safety.

Our overall complication rate of 23.6% is similar to the stated complication rate of 23.3% in the TeaM study which included data from 50 centres. In total, 5.2% (*n*=6) of our study cohort required unplanned surgery to treat complications.^6^ However, only three cases (2.6%) required surgery to treat complications within 90 days (3 months) of the index procedure. Our outcomes are therefore comparable with the TeaM study in which 2.8% of the study cohort required reoperations within 90 days (3 months) because of complications.^6^ In our study cohort, wound infection occurred in 11.4% despite the single-dose perioperative prophylactic antibiotic usage. Going forward, a more systematic approach, such as ‘Theatre Implant Checklist’ for surgical site infection prevention in implant-based breast surgery, may be required to reduce the infection rate in patients undergoing TM.^18^

Given the retrospective nature of the study, we were unable to obtain full data on other factors potentially contributing to complications such as body mass index and patient American Society of Anesthesiologists grade. Furthermore, T-junction wound healing may be influenced by preoperative ‘mark-up’ pattern and intraoperative technical aspects, which are beyond the scope of this retrospective study.^19^ There is also emerging use of negative pressure wound therapy in oncoplastic breast surgery, and further research is required to determine whether such therapy reduces the rate of T-junction wound-healing issues.^20^

In our study cohort, the median duration to the receipt of adjuvant therapy was 54 days. This is within the specified 56 days and reflected by our relatively low LRR and favourable DFS and OS.^12^ We found that wound complications resulted in a median delay of 21 days to the commencement of adjuvant therapy. This is mainly because of delayed wound healing or resolution of infection post-TM, which are mostly managed with non-operative treatments. We found that this modest delay did not impact survival outcomes. However, given our relatively modest sample size, future studies are required to further evaluate this research question and validate our findings. This is important given the increased utilisation of oncoplastic breast surgery in which the greater magnitude of surgery and higher complication rates need to be balanced against oncological principles that should not be compromised.

## Conclusions

Our study results demonstrate the oncological safety of TM with low LRR and favourable associated survival outcomes. Wound complications were mostly minor and managed conservatively. Although patients with wound complications had modest delay to the initiation of adjuvant therapy, their long-term oncological outcomes were comparable with those of patients who did not suffer from wound complications. Therefore, advanced BCS techniques such as TM should continue to be offered and utilised widely when clinically appropriate.
